# Increased Right Frontal Brain Activity During the Mandarin Hearing-in-Noise Test

**DOI:** 10.3389/fnins.2020.614012

**Published:** 2020-12-17

**Authors:** Fengxiang Song, Yi Zhan, James C. Ford, Dan-Chao Cai, Abigail M. Fellows, Fei Shan, Pengrui Song, Guochao Chen, Sigfrid D. Soli, Yuxin Shi, Jay C. Buckey

**Affiliations:** ^1^Department of Radiology, Shanghai Public Health Clinical Center, Fudan University, Shanghai, China; ^2^Space Medicine Innovations Laboratory, Geisel School of Medicine at Dartmouth, Hanover, NH, United States; ^3^Department of Psychiatry, Dartmouth-Hitchcock, Lebanon, NH, United States; ^4^House Clinic, Los Angeles, CA, United States

**Keywords:** central auditory processing, functional MRI, frontal lobe, tonal language, hearing-in-noise test

## Abstract

**Purpose:**

Previous studies have revealed increased frontal brain activation during speech comprehension in background noise. Few, however, used tonal languages. The normal pattern of brain activation during a challenging speech-in-nose task using a tonal language remains unclear. The Mandarin Hearing-in-Noise Test (HINT) is a well-established test for assessing the ability to interpret speech in background noise. The current study used Mandarin HINT (MHINT) sentences and functional magnetic resonance imaging (fMRI) to assess brain activation with MHINT sentences.

**Methods:**

Thirty native Mandarin-speaking subjects with normal peripheral hearing were recruited. Functional MRI was performed while subjects were presented with either HINT “clear” sentences with low-level background noise [signal-to-noise ratio (SNR) = +3 dB] or “noisy” sentences with high-level background noise (SNR = −5 dB). Subjects were instructed to answer with a button press whether a visually presented target word was included in the sentence. Brain activation between noisy and clear sentences was compared. Activation in each condition was also compared to a resting, no sentence presentation, condition.

**Results:**

Noisy sentence comprehension showed increased activity in areas associated with tone processing and working memory, including the right superior and middle frontal gyri [Brodmann Areas (BAs) 46, 10]. Reduced activity with noisy sentences was seen in auditory, language, memory and somatosensory areas, including the bilateral superior and middle temporal gyri, left Heschl’s gyrus (BAs 21, 22), right temporal pole (BA 38), bilateral amygdala-hippocampus junction, and parahippocampal gyrus (BAs 28, 35), left inferior parietal lobule extending to left postcentral gyrus (BAs 2, 40), and left putamen.

**Conclusion:**

Increased frontal activation in the right hemisphere occurred when comprehending noisy spoken sentences in Mandarin. Compared to studies using non-tonal languages, this activation was strongly right-sided and involved subregions not previously reported. These findings may reflect additional effort in lexical tone perception in this tonal language. Additionally, this continuous fMRI protocol may offer a time-efficient way to assess group differences in brain activation with a challenging speech-in-noise task.

## Introduction

Understanding speech in background noise is a challenging task involving multiple brain areas, including regions involved in auditory processing, interpreting language, maintaining attention, and using working memory (Bronkhorst and Plomp, 1992; Narayan et al., 2007; Shetake et al., 2011; Moore et al., 2014). The Hearing-in-Noise Test (HINT) is commonly used as a functional test of hearing ability, including central auditory function. Previous functional magnetic resonance imaging (fMRI) studies have revealed brain activity in both temporal and frontal areas during speech comprehension (Davis and Johnsrude, 2003; Wong P. C. M. et al., 2008; Adank et al., 2012). Wong P. C. M. et al. (2008) presented English words in speech-on-speech background noise with various signal-to-noise ratios (SNRs, +20 dB or −5 dB), and reported elevated brain activity for words in high-level noise in the left posterior superior temporal gyrus (STG) and left anterior insula. Davis and Johnsrude (2003) presented English sentences with added speech-spectrum noise (SNRs = −1 dB, −4 dB, or −6 dB). Distorted speech elicited an increased response in the left hemisphere, including parts of the STG, middle temporal gyrus (MTG), inferior frontal gyrus (IFG), precentral gyrus, and thalamus. Adank et al. (2012) investigated spoken sentence comprehension in Dutch, and revealed more activity in bilateral IFG and frontal operculum (FO) during noisy (SNRs = +2 dB, 0 dB, or −2 dB) speech processing than clear speech processing, although less activity was found in primary auditory and language networks. These results suggest that, in addition to the temporal areas involved in auditory and language processing, frontal areas become increasingly activated with higher levels of background noise during speech processing (Adank et al., 2012): increased frontal activation, especially in the left IFG and FO, were consistently found when additional noise processing was required in speech comprehension.

Although the previously mentioned fMRI studies have revealed consistent frontal activation patterns when comprehending speech with added background noise, few studies have investigated brain modulations during speech-in-noise processing in a tonal language. Hwang et al. (2006) examined brain activation of native Mandarin speakers when listening to narratives both with low-level white noise (SNR = +5 dB) and without noise. In contrast to previous findings, an overall decrease in brain activity was found in the low-level noise condition within the auditory, language, and memory networks, including in the left STG, MTG, parahippocampal gyrus (PHG), cuneus and thalamus, and the right STG, IFG, lingual gyrus, uncus, and fusiform gyrus. No increased brain activation was found in either temporal or frontal areas when comprehending noisy narratives. The inconsistent findings in frontal activation between the Hwang study and other studies might derive from the robustness of lexical tone perception in noise (Wang and Xu, 2020). Since the Hwang study used intelligible speech with a relatively high SNR and a passive listening paradigm, however, it is also possible that one or both of these factors contributed to the observed decrease in frontal activation.

In the current study, we aimed to examine brain activation during processing of noisy speech in a tonal language using spoken sentences from the Mandarin HINT (MHINT). A modified version of MHINT was administered during fMRI scanning in which word recognition was explicitly required. The SNRs (+3 dB, −5 dB) of background noise were manipulated to affect intelligibility. Brain activation differences were expected in cortical areas recruited for tone perception, in addition to previously reported regions for speech comprehension of speech in noise.

## Materials and Methods

### Subjects

Thirty healthy native Mandarin speakers participated in the current study. Three subjects without valid behavioral responses and five subjects with large head motions were excluded from further analysis. The remaining 22 subjects (seven female) were at a mean [± standard deviation (SD)] age of 32 ± 10.0 years with an average education of 16 ± 2.6 years. All participants were right-handed (confirmed by Edinburgh Handedness Inventory – Short Form), with normal peripheral hearing tests (<20 dB hearing level at the frequencies of 500, 1000, 2000, and 4000 Hz), and with no history of oral or written language impairment, or neurological or psychiatric diseases. All subjects had normal cognitive ability as revealed in the Montreal Cognitive Assessment. Coffee and smoking were prohibited for 3 h prior to scanning. The study was carried out in accordance with The Code of Ethics of the World Medical Association and was approved by the Ethics Committee of the Shanghai Public Health Clinical Center and the Committee for the Protection of Human Subjects at Dartmouth College. Written informed consent was obtained from all participants.

### Materials

We selected 120 sentences from the MHINT (Soli and Wong, 2008; Wong L. L. N. et al., 2008). All sentences were created by a linguistic expert and recorded in advance. The duration of sentences varied but were all less than 3 s. Noisy sentences were created by placing each pre-recorded sentence in the middle of a 3 s white noise interval, resulting in equal length stimuli even though sentence duration differed. To make the “clear” condition perceptually more comparable to the “noisy” condition, we added low level noise (+3 dB) to each pre-recorded sentence so that both “clear” and “noisy” sentences had some noise in the 3 s background that started slightly prior to the beginning of each sentence. To determine an appropriate SNR for the noisy sentences, we tested ten native Mandarin speakers at three different SNRs (−2 dB, −3.5 dB, and −5 dB) using 40 sentences for each SNR. This pilot study was carried out in a sound booth where auditory sentences with added noise were presented via Sennheiser HDA 200 supra-aural headsets calibrated with a flat-plate coupler. The results suggested −5 dB as the appropriate SNR for the “noisy” sentences, which provided an accuracy around 75%. To assess whether MR scanner noise would affect accuracy, another ten more native speakers with normal hearing were tested with the scanner on and off. A set of insert earphones (S15, Sensimetrics) and an MR compatible headset (S9900, Shenzhen Sinorad Medical Electronics Inc., China) were used to reduce scanner noise. The average accuracy with the scanner on and off was 72% and 75%, respectively.

**FIGURE 1:**
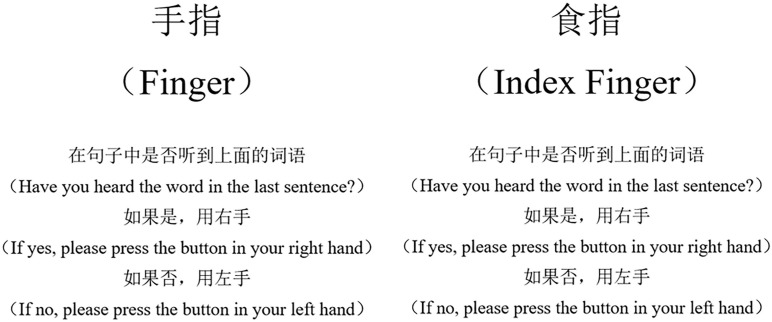
The target **(left)** and foil **(right)** words for a sample sentence. The word-for-word translation of the sample sentence was, “She was cutting vegetables when she accidentally cut her finger (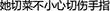).” The target and foil words are similar in duration in Mandarin.

For each sentence, we selected a noun as the “target” word and paired it with a “foil” word. The two words were similar in duration and were equally plausible in the context. Each sentence was auditorily presented through the headset, and then either the target or foil word was presented visually on a computer screen. The subjects were required to indicate whether the visually presented word was included in the sentence (target) or not (foil) via a button press (Figure 1). Participants were instructed to decide as quickly as possible. To improve experimental compliance during the fMRI scan, a training session was given to participants before scanning to ensure they understood the task. The training session included five sentences distinct from the formal task and was conducted on a laptop. Materials used in the scanning session were listed in Supplementary Table 1, including the corresponding target and foil words, their English translations, and phonetic transcriptions.

### Task Design

For statistical analysis of brain activation, we used an event-related design. The stimulus set consisted of 40 MHINT sentences, with 30% presented at a high SNR level (+3 dB, clear condition) and the remaining presented at a low SNR level (−5 dB, noisy condition). In both clear and noisy conditions, there were an equal number of presentations of target and foil words. Furthermore, there were three 18 s rest periods, during which the word “rest” was displayed on the screen (rest condition). Intervals for the three conditions were ordered for optimal efficiency and counterbalanced using a randomization approach implemented in the opseq2 program (Dale, 1999; Davis et al., 2011). Presentation schedules were calculated in optseq2 using a time increment of 3 s and a post-stimulus window of 12 s. Between events, there were gaps with varying intervals.

### MRI Acquisition

The subject was placed in the scanner and was able to view a screen through a mirror attached to the head coil. The stimulus was presented via a Visual and Audio Stimulation System for fMRI (S9900, Shenzhen Sinorad Medical Electronics Inc., China). Imaging data were acquired using a 3.0 T Philips Ingenia system (Philips Healthcare, Best, Netherlands). To decrease the background noise generated by the scanner, a sound attenuating headset (S9900, Shenzhen Sinorad Medical Electronics Inc., China) and insert earpieces (S15, Sensimetrics) were used. Foam inserts were used to reduce head motion.

The main scanning parameters were as follows. A T1-weighted high-resolution structural scan was acquired: 170 slices, repetition time (TR) = shortest (8.1–8.2 ms), echo time (TE) = shortest (3.7–3.8 ms), field of view (FOV) = 240 mm × 225 mm, matrix = 384 × 384, slice thickness = 1 mm. Next, an echo planar imaging (EPI) sequence was obtained using TR/TE = 3000/25 ms, slice thickness = 4 mm, gap = 0 mm, flip angle = 75°, FOV = 224 mm × 224 mm, matrix = 128 × 128, voxel size = 3.4 mm × 3.4 mm × 4.0 mm, 34 slices. The EPI sequence took 9 min and 9 s including a 15 s pre-scan period to ensure a stable signal. The pre-scan period was not included in the data processing. The total scanning time was 15 min and 9 s.

### fMRI Data Analyses

Image pre-processing and statistical analyses were conducted using the Statistical Parametric Mapping software package (SPM12, Wellcome Centre for Human Neuroimaging, United Kingdom). The pre-processing steps included slice-timing correction, realignment, co-registration to structural images, and spatial normalization to the MNI152 standard template. The preprocessed data were resampled into 2 mm cubic voxels and spatially smoothed with a 8 mm × 8 mm × 8 mm full width at half-maximum (FWHM) isotropic Gaussian kernel. Drift of the volumes was removed from the smoothed files using the LMGS approach (Macey et al., 2004). We further identified problematic volumes in each scan using Artifact Detection Tools^1^. Specifically, a volume was defined as an outlier if the head displacement in any direction was greater than 1 mm from the previous frame, or if the global mean intensity in the volume was greater than 3 SDs from the mean image intensity of the entire scan. Each outlier volume was modeled in a binary covariate. Five subjects with more than 10 outlier volumes were excluded from further analysis.

For the first-level analyses, a general linear model was estimated using the preprocessed images. Time blocks for the auditory presentations of clear and noisy sentences plus their associated response windows were included as conditions. Six head motion parameters as well as binary variables indicating problematic volumes were included as covariates. For the model estimation, the parameters were evaluated and calculated using the default restricted maximum likelihood method in SPM12. The main contrast was “noisy” versus “clear”; therefore, the positive and negative values in group differences indicate increased (noisy > clear) and decreased (noisy < clear) brain activation in processing of the more noisy speech, respectively. Significance of group-level differences in the noisy versus clear contrast was evaluated in a second-level analysis using one-sample *t*-tests with age and gender as covariates. Voxel and cluster results were thresholded at a *P* < 0.05 significance level with family wise error (FWE) correction, with a cluster-defining threshold of *P* = 0.001. Similar models were used to determine group activation patterns during the noisy and clear conditions relative to the rest condition.

## Results

### Behavioral Results

**FIGURE 2:**
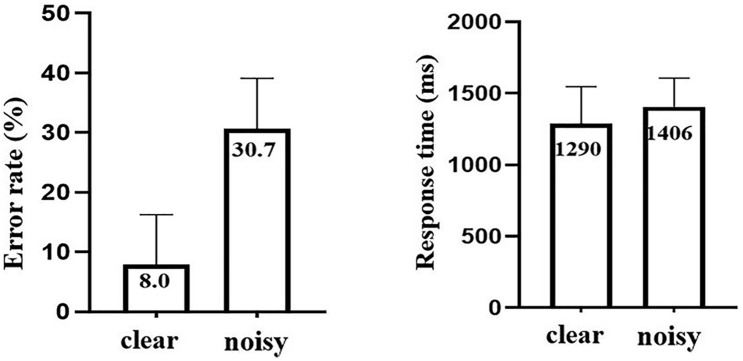
The error rates and response times in the clear and noisy conditions. The error bars indicate SD.

The behavioral results are shown in Figure 2. Participants were significantly less accurate (*t* = 9.66, *P* < 0.05) and significantly slower in the noisy condition (*t* = 3.00, *P* < 0.05). The mean (±SD) error rate was 8.0% (±8.3%) in the clear and 30.7% (±8.4%) in the noisy condition. The mean response time was 1290 ms (±257.1 ms) in the clear and 1406 ms (±201.6 ms) in the noisy condition.

### Neuroimaging Results

#### Noisy versus Clear

**FIGURE 3:**
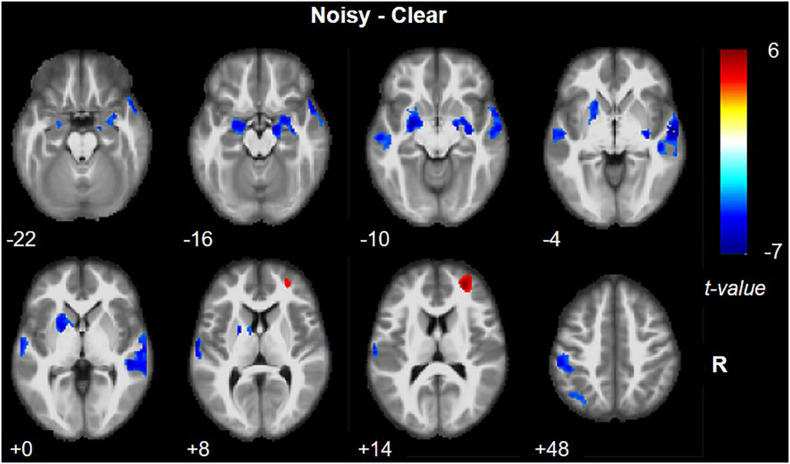
Brain activation in noisy speech processing in contrast to clear speech processing. Red and blue colors denote increased and decreased activation in the noisy condition, respectively.

Brain activation during noisy speech processing in contrast to clear speech processing is presented in Figure 3 and Table 1. Larger brain activity in the right superior frontal gyrus (SFG) extending to the right middle frontal gyrus (MFG) (BAs 46 and 10) was observed in the noisy condition. Reduced activation was seen in secondary auditory, language, auditory, memory, and somatosensory areas, including bilateral STG (BA 22) and MTG (BA 21), left Heschl’s gyrus (BAs 41 and 42), right temporal pole (BA 38), bilateral amygdala-hippocampus junction and PHG (BAs 28 and 35), left inferior parietal lobule extending to left postcentral gyrus, and putamen (BA 40).

**TABLE 1:** Brain activation in noisy speech processing.

	Brain region	BA	Hemisphere	Cluster size	Peak voxel coordinate	*t*-value
Noisy > Clear	SFG	46	R	406	28, 50, 14	5.895
Clear > Noisy	STG	22	R	1024	60, −12, −4	−7.052
	Amygdala	34	L	673	−26, −4, −10	−6.657
	STG	22	L	466	−62, −12, 10	−6.531
	PHG	35	R	460	16, −10, −16	−6.088
	IPL	40	L	783	−42, −46, 58	−5.656

*BA, Brodmann area; SFG, superior frontal gyrus; STG, superior temporal gyrus; PHG, parahippocampal gyrus; IPL, inferior parietal lobe.*

#### Noisy/Clear versus Rest

**FIGURE 4:**
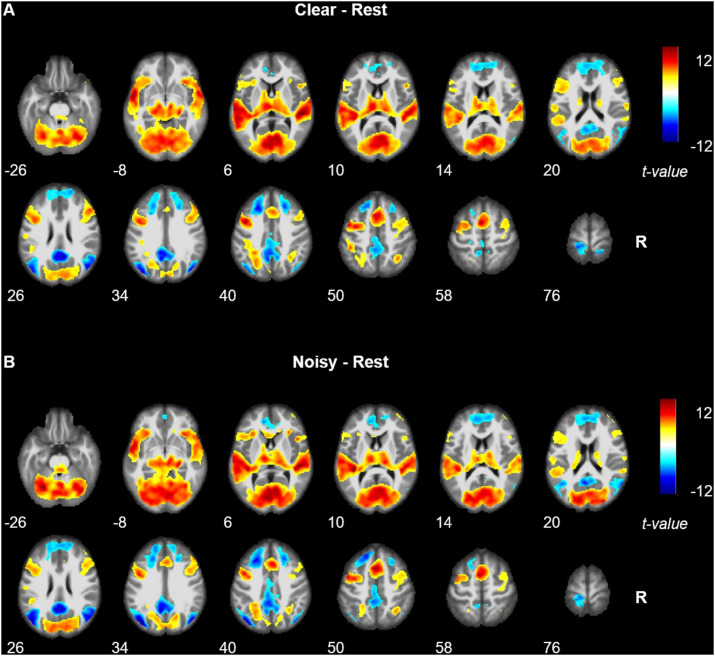
Brain activation revealed by clear versus rest contrast **(A)** and noisy versus rest contrast **(B)**. Red and blue colors denote increased and decreased activation in speech comprehension conditions, respectively.

For the clear versus rest contrast, most of the activation (Figure 4A) was clustered at primary and secondary auditory and visual cortex, including bilateral STG (BA 22) and MTG (BA 21), bilateral Heschl’s gyrus (BAs 41, 42), and bilateral inferior and middle occipital gyrus, lingual gyrus, fusiform gyrus, calcarine gyrus, and cuneus (BAs 17, 18, and 19). We also noted significant activation in the cerebellum. Other areas activated included bilateral temporal pole (BA 38), bilateral IFG (BA 47), bilateral posterior medial frontal gyrus, bilateral precentral and postcentral gyrus (BA 6), bilateral insula (BA 48), bilateral thalamus, left putamen, left caudate nucleus, and bilateral middle cingulate cortex (BA 32). For the noisy versus rest contrast, the activation was mostly the same except there was no activation of right postcentral and precentral gyri (Figure 4B).

During both clear and noisy sentence processing, brain activations were significantly reduced in areas associated with the default mode network, including bilateral MFG and SFG, superior medial gyrus, middle orbital gyrus, rectal gyrus, precuneus, paracentral lobe, middle and posterior cingulate cortex, calcarine gyrus, MTG, angular gyrus, middle occipital gyrus, and postcentral gyrus.

## Discussion

This study calculated brain activation patterns during processing of Mandarin with background noise using a modified version of the MHINT task. Increased activation of the right frontal lobe including SFG (BA 46) and MFG (BA 10) were related to a higher level of background noise during speech comprehension. The current finding of increased frontal lobe activation during noisy speech processing is consistent with the previous Adank et al. (2012) study, although it was in the bilateral IFG and FO in that work. The difference in brain areas activated between studies might reflect the different effects of background noise during tonal and non-tonal language comprehension. In a meta-analysis of fMRI in tonal languages, right MFG was reported as one of the main brain areas responsible for sentence-level prosody processing, and was closely related to tone processing (Liang and Du, 2018). Right SFG was also related to tonal processing (Xu et al., 2013). When processing Chinese sentences lacking tonal information, enhanced activation in the right SFG was observed. Although the role of middle and superior frontal areas during speech-in-noise recognition remains to be further investigated, the current findings support, but cannot fully confirm, the hypothesis of increased or additional tone processing in tonal language recognition when there is background noise (Wang and Xu, 2020).

In addition, the right SFG and MFG (BAs 46, 10) are both related to working memory (Leung et al., 2002; Pochon et al., 2002; Zhang et al., 2003), and the SFG in particular is responsible for phonological working memory (Heim et al., 2003). Prior studies also discussed the reliance on working memory capacity in processing speech in background noise. Michalek et al. (2018) recruited ninety six participants to examine the effects of SNR and working memory capacity on speech recognition. They found that working memory capacity mediated speech processing in noise, and that reliance on working memory capacity increased as the noise level increased. The HINT paradigm might engage more “working memory” than other previously used paradigms such as speech comprehension and passive listening, as it requires the participants to remember the exact words in the sentences they hear. It is possible that right frontal areas are more engaged in HINT than other paradigms and become more activated when background noise is present.

The current study also showed reduced activation during noisy speech comprehension in several brain networks, including the central auditory, language, memory, and sensorimotor cortex, which is consistent with some previous studies (Hwang et al., 2006; Adank et al., 2012) but inconsistent with others (Wong P. C. M. et al., 2008; Davis et al., 2011). This disagreement may be attributable to the nature of background noise (Adank et al., 2012): fluctuating or changing background sounds such as multi-speaker babble used in Wong P. C. M. et al.’s (2008) study and bursts of noise used in Davis et al.’s (2011) study might lead to additional sound processing and therefore increased activation in core auditory and language networks, while in contrast, steady background noise as used in Adank et al.’s (2012) and the current study plays a role as an energetic masker reducing speech intelligibility, covering or supressing auditory speech cues, leading to a reduction of relevant brain activities.

The present study has some limitations. First, we did not directly compare brain modulation of noise between a tonal and non-tonal language. The current finding of increased activation in right SFG and MFG therefore could be linked to factors other than tone processing. Future studies investigating both tonal and non-tonal language using the same speech-in-noise paradigm are needed. Second, we employed a new speech-in-noise paradigm (the MHINT sentences) which differed from those in previous fMRI studies. The activation of right frontal subregions in the current study could have been partially influenced by the paradigm, which needs further validation in future studies. However, the current findings of brain activation during either noisy or clear sentence processing in contrast to the resting state are similar to those observed in the Hwang study (Hwang et al., 2006), indicating that the MHINT paradigm activated the core brain network of speech comprehension much like other paradigms such as passive listening. The current paradigm also has several advantages. The MHINT is widely available in many languages. It is short and commonly used in clinical settings to assess the speech-in-noise processing deficits in patients with brain conditions such as the human immunodeficiency virus infection (Zhan et al., 2018). Third, the use of a continuous fMRI paradigm means that scanner noise could be a further source of acoustic stimulation: although the scanner noise should have been effectively subtracted from the contrasts of interest, it may have influenced the overall activation strength. Despite this, results similar to previous studies using sparse scanning were seen, and the use of continuous scanning allowed for much greater time efficiency. Fourth, the number of enrolled cases was small, although relatively larger than previous studies. Future studies using a larger sample size are needed.

### Conclusion

Increased activation in the right superior and middle frontal gyrus (BAs 46,10) were revealed in background noise processing during Mandarin speech comprehension. This was accompanied by reduced activation in primary auditory areas. These two frontal sub-regions are relevant for tone perception and phonological working memory and have not been reported in previous speech-in-noise studies using non-tonal languages. These findings suggest additional frontal activation related to processing of lexical tones and prosody during MHINT compared to activations induced by English language stimuli in background noise.

## Data Availability Statement

The raw data supporting the conclusions of this article will be made available by the authors, without undue reservation.

## Ethics Statement

The studies involving human participants were reviewed and approved by the Ethics Committee of the Shanghai Public Health Clinical Center and the Committee for the Protection of Human Subjects at Dartmouth College. The patients/participants provided their written informed consent to participate in this study.

## Author Contributions

FNS and YZ: protocol development, data collection, and data analysis. JF: protocol development and MR analysis. D-CC: data collection and data analysis. AF: protocol development, HINT sentence evaluation, and data analysis. FIS, PS, and GC: data collection. SS: HINT interpretation. YS and JB: study conceptualization, project administration, protocol development, and data analysis. All authors were involved in interpreting the results and writing the manuscript.

## Conflict of Interest

The authors declare that the research was conducted in the absence of any commercial or financial relationships that could be construed as a potential conflict of interest.

## Footnotes

1 http://www.nitrc.org/projects/artifact_detect/

## Supplementary Material

Supplementary Table 1 Target and foil words used in the experiment.
