# Corrigendum: Increased Right Frontal Brain Activity During the Mandarin Hearing-in-Noise Test

**DOI:** 10.3389/fnins.2021.696057

**Published:** 2021-05-12

**Authors:** Fengxiang Song, Yi Zhan, James C. Ford, Dan-Chao Cai, Abigail M. Fellows, Fei Shan, Pengrui Song, Guochao Chen, Sigfrid D. Soli, Yuxin Shi, Jay C. Buckey

**Affiliations:** ^1^Department of Radiology, Shanghai Public Health Clinical Center, Fudan University, Shanghai, China; ^2^Space Medicine Innovations Laboratory, Geisel School of Medicine at Dartmouth, Hanover, NH, United States; ^3^Department of Psychiatry, Dartmouth-Hitchcock, Lebanon, NH, United States; ^4^House Clinic, Los Angeles, CA, United States

**Keywords:** central auditory processing, functional MRI, frontal lobe, tonal language, hearing-in-noise test

In the original article, we neglected to include the funder Shanghai Municipal Commission of Health and Family Planning, No. 201840146 to YS. The updated funding statement therefore reads:

This work was supported by the National Institutes of Health grant from the National Institute of Neurological Disorders and Stroke (R01NS108809) and the Shanghai Municipal Commission of Health and Family Planning (No. 201840146).

The authors apologize for this error and state that this does not change the scientific conclusions of the article in any way. The original article has been updated.

