# Supplementary material for: Increased Right Frontal Brain Activity During the Mandarin Hearing-in-Noise Test
Source: Front Neurosci. 2020 Dec 17;14:614012. doi: 10.3389/fnins.2020.614012 (PMC7773781; doi:10.3389/fnins.2020.614012)
Supplement: Supplementary Table 1 — Target and foil words used in the experiment. [file Data_Sheet_1.PDF]

Table S1. Target and foil words used in the experiment

Noisy sentences + foil words			Noisy sentences	
	Target word	Foil word		Target word
1	ping1 pang1 qiu2<br>乒 乓 球<br>table tennis	yu3 mao2 qiu2<br>羽 毛 球<br>badminton	21	jue2 sai4<br>决 赛<br>final
2	yan1 hui1 gang1<br>烟 灰 缸<br>ashtray	la1 ji1 tong3<br>垃 圾 桶<br>trash can	22	shou1 yin1<br>收 音<br>radio
3	qi4 che1 zhan4<br>汽 车 站<br>bus stop	huo3 che1 zhan4<br>火 车 站<br>train station	23	wo3 men1<br>我 们<br>we
4	gong1 zuo4<br>工 作<br>work	xue2 xi2<br>学 习<br>study	24	fei1 die2<br>飞 碟<br>flying saucer
5	san4 bu4<br>散 步<br>a walk	pao3 bu4<br>跑 步<br>a run	25	zhi1<br>支<br>piece
6	ba4 ba0<br>爸 爸<br>Dad	ye3 ye0<br>爷 爷<br>Grandpa	26	chun1 tian1<br>春 天<br>spring
7	jin1 tian1<br>今 天<br>today	zuo2 tian1<br>昨 天<br>yesterday	27	na3 ge4<br>哪 个<br>which
8	zhuo1 zi0<br>桌 子<br>table	yi3 zi0<br>椅 子<br>chair	28	qian2<br>钱<br>money
9	xiao3 hai2<br>小 孩<br>child	xiao3 gou3<br>小 狗<br>puppy	29	men2 kou3<br>门 口<br>door
10	na4 ge0<br>那 个<br>that	zhe4 ge0<br>这 个<br>this	30	jin1 nian2<br>今 年<br>this year
11	zuo4 wen2<br>作 文<br>composition	shu1 fa3<br>书 法<br>calligraphy	31	liu2 gan3<br>流 感<br>flu
12	tong2 xue2<br>同 学<br>classmates	peng2 you3<br>朋 友<br>friends	32	jie3 fu1<br>姐 夫<br>brother-in-law
13	xia4 wu3<br>下 午<br>afternoon	wan3 shang4<br>晚 上<br>evening	33	peng2 you3<br>朋 友<br>kid
14	jia4 shi3<br>驾 驶<br>driving	lv4 shi1<br>律 师<br>lawyer's	34	guo2 wai4<br>国 外<br>abroad

Clear sentences + foil words			Clear sentences	
	Target word	Foil word		Target word
15	su4 she4<br>宿 舍<br>dormitories	jiao4 shi4<br>教 室<br>classrooms	35	shou3 zhi3<br>手 指<br>finger
16	xia4 ban1<br>下 班<br>off work	shang4 ban1<br>上 班<br>going to work	36	xian4 cheng2<br>县 城<br>country seat
17	xiao3 mao1<br>小 猫<br>kitten	xiao3 gou3<br>小 狗<br>puppy	37	jiang3 hua3<br>讲 话<br>spoke
18	zhe4 ge0<br>这 个<br>this	na4 ge0<br>那 个<br>that	38	ba4 ba0<br>爸 爸<br>Dad
19	fa2 wei4<br>乏 味<br>boring	wu2 qu4<br>无 趣<br>uninteresting	39	jian4 zhu4<br>建 筑<br>construction
20	pai2 zi0<br>牌 子<br>brand	xing2 hao4<br>型 号<br>model	40	nv3 hai2<br>女 孩<br>girl
